# Association between preoperative evaluation with lung ultrasound and outcome in frail elderly patients undergoing orthopedic surgery for hip fractures: study protocol for an Italian multicenter observational prospective study (LUSHIP)

**DOI:** 10.1186/s13089-021-00230-w

**Published:** 2021-06-07

**Authors:** Luigi Vetrugno, Enrico Boero, Elena Bignami, Andrea Cortegiani, Santi Maurizio Raineri, Savino Spadaro, Federico Moro, Stefano D’Incà, Loris D’Orlando, Felice Eugenio Agrò, Mattia Bernardinetti, Francesco Forfori, Francesco Corradi, Sandro Pregnolato, Mario Mosconi, Valentina Bellini, Federico Franchi, Pierpaolo Mongelli, Salvatore Leonardi, Clemente Giuffrida, Marco Tescione, Andrea Bruni, Eugenio Garofalo, Federico Longhini, Gianmaria Cammarota, Edoardo De Robertis, Giuseppe Giglio, Felice Urso, Tiziana Bove, The LUSHIP Study Investigators, Lisa Mattuzzi, Nicola Federici, Silvia Delrio, Francesco Meroi, Luca Flaibani, Clara Zaghis, Daniele Orso, Serena Tomasino, Bruno Dottore, Michele Divella, Sabrina Mussetta, Gaia Musso, Angela Minunno, Carlo Barbero, Mattia Puppo, Francesco Saturno, Alberto Nicolò Galvano, Mariachiara Ippolito, Leo Massari, Margherita Bianconi, Gaetano Caruso, Riccardo Ragazzi, Carlo Alberto Volta, Silvia Mongodi, Francesco Mojoli, Filippo Riccone, Sabino Scolletta, Sebastiano Macheda, Serafino Vulcano, Giovanni Cosco, Eugenio Vadalà, Erika Taddei, Alessandro Isirdi

**Affiliations:** 1 Department of Medicine, University of Udine, Via Colugna no. 50, 33100 Udine, Italy; 2 University-Hospital of Friuli Centrale, ASFC, P.le S. Maria della Misericordia no. 15, 33100 Udine, Italy; 3 Anesthesia and Intensive Care Unit, San Giovanni Bosco Hospital, Turin, Italy; 4 Anesthesiology, Critical Care and Pain Medicine Division, Department of Medicine and Surgery, University of Parma, Parma, Italy; 5 Department of Surgical, Oncological and Oral Science (Di.Chir.On.S), University of Palermo, Palermo, Italy; 6 Department of Anesthesia Intensive Care and Emergency, Policlinico Paolo Giaccone, Palermo, Italy; 7 Department of translational medicine, Anesthesia and Intensive Care, University of Ferrara, Ferrara, Italy; 8 Department of Medicine, Unit of Anesthesia Intensive Care Pain Management, Università Campus Bio-Medico Di Roma, Rome, Italy; 9 Department of Surgical, Medical and Molecular Pathology and Critical Care Medicine, University of Pisa, Pisa, Italy; 10 Department of Anesthesia and Intensive Care, Ente Ospedaliero Ospedali Galliera, Genova, Italy; 11 Department of Clinical-Surgical, Diagnostic and Pediatric Sciences, University of Pavia, Pavia, Italy; 12 Orthopedics and Traumatology, Fondazione IRCCS Policlinico San Matteo, Pavia, Italy; 13 Department of Medicine, Surgery and Neuroscience, Anesthesiology and Intensive Care, University of Siena, Siena, Italy; 14 IRCCS Centro Neurolesi Bonino-Pulejo, Messina, Italy; 15 Anesthesia and Intensive Care Unit, Grande Ospedale Metropolitano, Reggio Calabria, Italy; 16 Anesthesia and Intensive Care Unit, Department of Medical and Surgical Science, Magna Graecia University, Catanzaro, Italy; 17 Section of Anaesthesia, Analgesia, and Intensive Care, Department of Medicine and Surgery, University of Perugia, Perugia, Italy

## Abstract

**Background:**

Hip fracture is one of the most common orthopedic causes of hospital admission in frail elderly patients. Hip fracture fixation in this class of patients is considered a high-risk procedure. Preoperative physical examination, plasma natriuretic peptide levels (BNP, Pro-BNP), and cardiovascular scoring systems (ASA-PS, RCRI, NSQIP-MICA) have all been demonstrated to underestimate the risk of postoperative complications. We designed a prospective multicenter observational study to assess whether preoperative lung ultrasound examination can predict better postoperative events thanks to the additional information they provide in the form of “indirect” and “direct” cardiac and pulmonary lung ultrasound signs.

**Methods:**

LUSHIP is an Italian multicenter prospective observational study. Patients will be recruited on a nation-wide scale in the 12 participating centers. Patients aged  >  65 years undergoing spinal anesthesia for hip fracture fixation will be enrolled. A lung ultrasound score (LUS) will be generated based on the examination of six areas of each lung and ascribing to each area one of the four recognized aeration patterns—each of which is assigned a subscore of 0, 1, 2, or 3. Thus, the total score will have the potential to range from a minimum of 0 to a maximum of 36. The association between 30-day postoperative complications of cardiac and/or pulmonary origin and the overall mortality will be studied. Considering the fact that cardiac complications in patients undergoing hip surgery occur in approx. 30% of cases, to achieve 80% statistical power, we will need a sample size of 877 patients considering a relative risk of 1.5.

**Conclusions:**

Lung ultrasound (LU), as a tool within the anesthesiologist’s armamentarium, is becoming increasingly widespread, and its use in the preoperative setting is also starting to become more common. Should the study demonstrate the ability of LU to predict postoperative cardiac and pulmonary complications in hip fracture patients, a randomized clinical trial will be designed with the scope of improving patient outcome.

*Trial registration *ClinicalTrials.gov, NCT04074876. Registered on August 30, 2019.

**Supplementary Information:**

The online version contains supplementary material available at 10.1186/s13089-021-00230-w.

## Background

One of the most common orthopedic causes leading to hospital admission in frail elderly patients is hip fracture [1, 2]. According to the European Society of Cardiology (ESC) and the European Society of Anesthesiology (ESA) guidelines, hip surgery is associated with an intermediate level risk of complication, ranging between 1 and 5% [3, 4]. However, in elderly patients with limited physiological reserve, the incidence of complication is much higher, between 22 and 53%; thus hip fracture fixation should be considered a high-risk procedure in these patients [5]. Anesthesiologists are expected to assess the risk of these patients and to take the necessary steps to improve their outcome [6, 7]. Today, to quantify and predict the risk of perioperative morbidity and mortality in these patients, in particular of cardiac origin, the following classification systems are frequently used: the American Society of Anesthesiologists Physical Status (ASA-PS) [8] the revised cardiac risk index (RCRI) [9], and the National Surgical Quality Improvement Program Myocardial infarction and Cardiac Arrest (NSQIP-MICA) [10]. Unfortunately, the literature shows that these scores generally work only moderately well and do not accurately predict mortality risk [11]. The ESC/ESA and the American College of Cardiology and American Heart Association (ACC/AHA) guidelines recommend the evaluation of metabolic equivalents (METS) as an important tool for patient risk stratification [3, 4, 12]. But METS evaluation in older patients with many comorbidities is not feasible. Indeed, studies confirming the utility of METS, both self-reported and tested, in these patients are still lacking [13, 14]. The Canadian Cardiovascular Society guidelines for perioperative cardiac risk assessment and management in noncardiac surgery patients have included the use of natriuretic peptide assessment (brain natriuretic peptide [BNP] and its precursor pro-natriuretic peptide NT-pro-BNP natriuretic peptide) in their centripetal key decision tree model [15]. But, once again, in moderate and higher risk patients, the use of these screening tools has shown a high negative predictive value (NPV), whilst performing better in relation to low-risk patients [16, 17].

Lung ultrasound (LU) has become an indispensable tool within the Anesthesiologist’s diagnostic arsenal, and some reports have started to highlight its role in the perioperative setting for perioperative outcome evaluation [18–20]. It is, therefore, fundamental that the utility of this tool be investigated in a large patient population in much greater detail. Considering the fact that LU examination is based on the exploration of ultrasound artifacts (A-lines, B-lines, lung sliding, focal interstitial syndrome—i.e., multiple B-lines) and the consolidated parenchyma, as well as pleural effusion in patients with pneumonia [21], we hypothesize that this tool may provide better “direct” evidence of preoperative pulmonary status—also supported by the fact that the sensitivity and specificity of LU are both known to be superior to chest radiography, which provides relevant information in just 0.1% of cases [22]. Furthermore, through the detection of diffuse B-lines over different zones, LU permits us to make an “indirect” evaluation of the underlying cardiac status of patients suffering from cardiac disease [23, 24]. Both these characteristics of LU could also echo the American College of Physicians (ACP) recommendation that all patients with hip fracture should undergo a risk assessment that focuses on chronic obstructive pulmonary disease (COPD) and congestive heart failure [25]. The primary aim of the present study is to evaluate, in a large population of elderly patients undergoing hip fracture repair, whether a systematic preoperative LU examination is able to provide bedside real-time information pertaining to the patients’ underlying “indirect” cardiac and “direct” pulmonary statuses, and can be used to asses perioperative cardiac and pulmonary outcome. The second aim is to compare the data gathered in the form of the lung ultrasound score (LUS) with the data provided by the traditional risk scores for preoperative evaluation, i.e., by ASA-PS, RCRI, NSQIP-MICA.

## Methods

This study was approved by the Ethics Committee of Friuli Venezia Giulia (CEUR-FVG), being the coordinating center, with the identification number 2817, dated June 4, 2019. The study was also registered at clinicaltrials.gov (https://clinicaltrials.gov/ct2/show/NCT04074876), identifier: NCT04074876, dated August 30, 2019.

### Study design and patients

LUSHIP is an Italian multicenter prospective observational study (LUSHIP.it). Patients will be recruited on a nation-wide scale in the 12 participating centers, each of which received approval from their Institution’s Ethics Committee prior to the enrolment of the first patient. Inclusion criteria are: age  >  65 years; hip fracture needing urgent surgery (<  24 h); spinal anesthesia; informed patient consent to participate in the study. Exclusion criteria are: inability to obtain informed patient consent; acute heart failure at the time of preoperative evaluation, defined as a clinical syndrome characterized by typical symptoms: “breathlessness, ankle swelling, and fatigue, that may be accompanied by the following signs: elevated jugular venous pressure, pulmonary crackles and peripheral oedema, caused by a structural and/or functional cardiac abnormality, resulting in reduced cardiac output and/or elevated intracardiac pressures at rest or during stress” (2016 ESC Guidelines) [26]; recent major adverse cardiac events (MACE) defined as arrhythmia (atrial fibrillation/flutter), myocardial infarction, or cardiac arrest in the previous 6 months; a history of pre-existing pulmonary pathologies: known history of pulmonary fibrosis, chronic renal failure on dialysis, fibrothorax, recent pneumothorax, lobectomy or pneumonectomy.

### Protocol for lung ultrasound

**Fig. 1:**
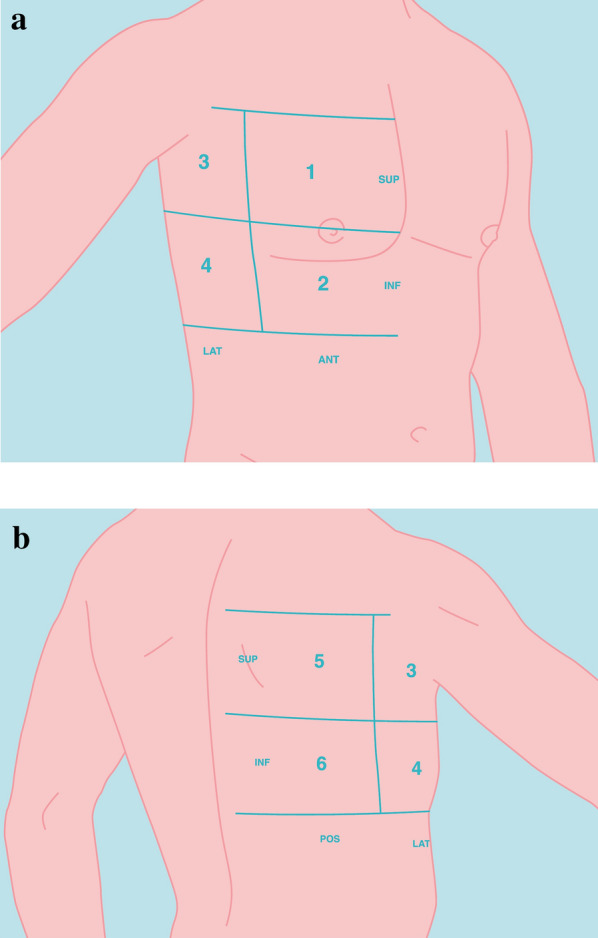
Each hemithorax is divided in six regions: two anterior, two lateral, and two posteriors, following to anatomical landmarks set by parasternal, anterior and posterior axillary lines. Each region is divided in superior and inferior. To perform an examination, adjacent intercostal spaces are explored in each region of interest, sliding the probe along intercostal space

**Fig. 2:**
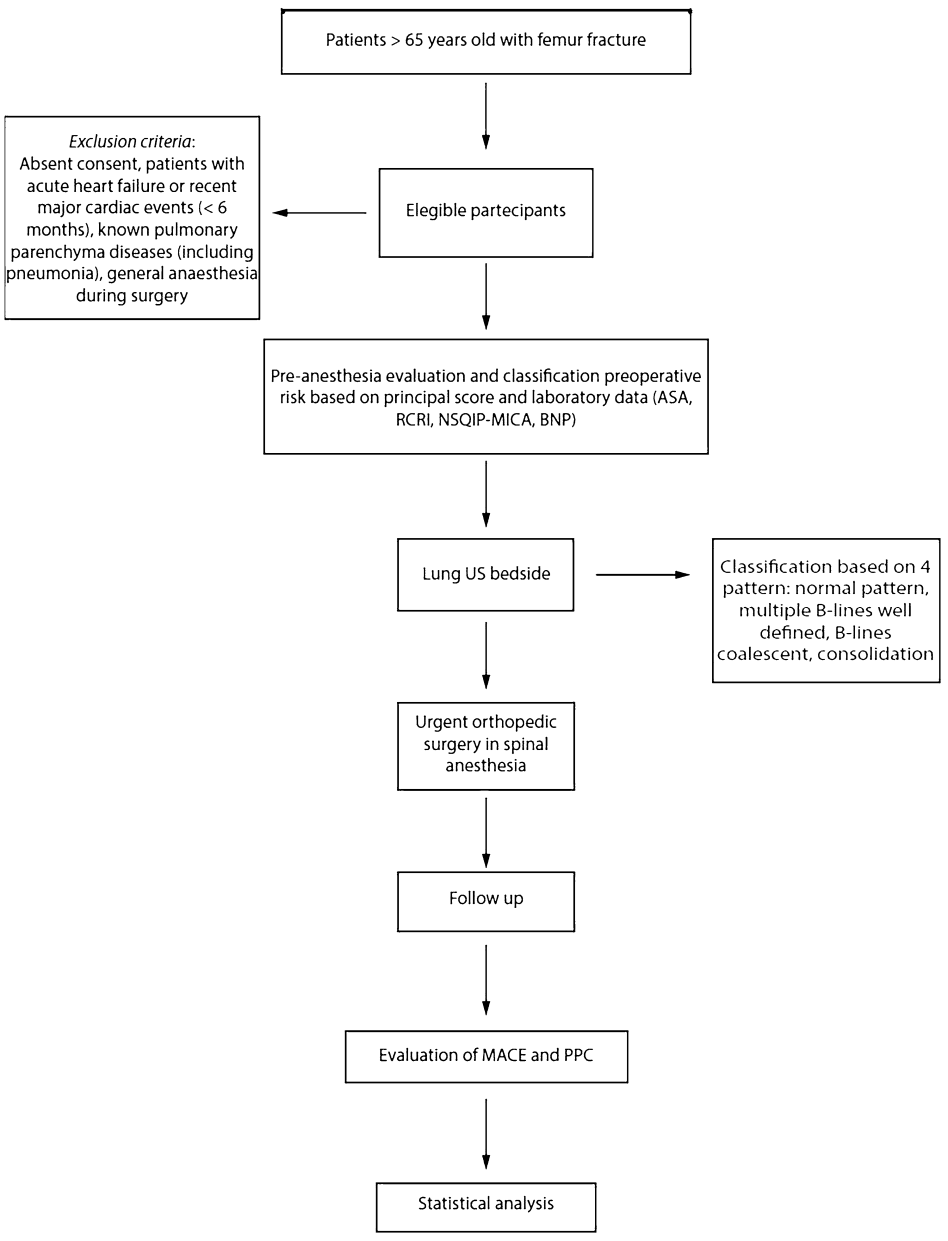
Study flow enrolment

The LUS recognizes four aeration patterns, and assigns to each of them a value ranging between 0 and 3. This evaluation is repeated in six areas of each lung. Using the anterior and posterior axillary line as vertical boundaries, the areas are divided into two anterior (superior and inferior), two lateral, and two posterior areas. The possible patterns are: (i) normal aeration—A-lines or less than two B-lines with lung sliding (score 0); (ii) moderate loss of aeration—three or more well-spaced B-lines with lung sliding (score 1); (iii) severe loss of aeration—coalescent B-lines with lung sliding (score 2); (iv) complete loss of aeration—tissue-like pattern or consolidation (score 3). The sum of all subscores will constitute the overall score. All adjacent intercostal spaces will be analyzed for each area by moving the probe through each space (Fig. 1) and diagram flow (Fig. 2). To standardize the evaluation of LU images, one of the authors (EBo) will coordinate the sharing by e-email of inter-reader agreement video-clip exams between the 12 centers, and the principal investigator for each Institution, plus two collaborators for each center. The ultrasound machine used in the primary center will be the GE Health-Care V-scan, but other machines available in each center could be used. Data collection will continue perioperatively and until hospital discharge for assessment of 30-day clinical deterioration, i.e., pulmonary, MACE, and mortality, defined according to the standards for definitions and use of outcome measures for clinical effectiveness research in perioperative medicine by the European Society of Anesthesia (ESA) and European Society of Intensive Care Medicine (ESICM) [27].

### Patient consent and data protection

Patients will receive information about the study, and written consent will be requested. In the case that the patient is unable write their signature, verbal consent will be asked in the presence of two testimonies. Patient data will be processed according to the Declaration of Helsinki and the European Privacy Regulation 2016/679 for General Data Protection Regulation (GDPR). The implementation of the study will not alter the management of the patient in any way during or following surgery. Each center is provided with an identical case report form (CRF) (Additional file 1). A principal investigator (PI) will be nominated for each participating center, who will be responsible for their institution’s data collection, ensuring proper concealment of patient identity on the linked CRF, and storing links between sensitive data and patient univocal codes under password protection. De-identified patient data will be uploaded onto a web platform by each PI, who will be provided with a personal username and password, thereby creating the study’s final database. The steering committee will consist of four investigators (LV, EB, AC, TB) plus six members with recognized expertise in LU (FC, FF, FM, SM, MB, EBo). In the case of any difficulties or problems, each PI will be able to communicate with the study’s other PI. Two independent investigators will perform data management activities on the database and check for abnormalities and inconsistencies. The study will be reported according to the Standards for the Reporting of Diagnostic Accuracy Studies (STARD) for Point-of-Care Ultrasound (POCUS) [28, 29]. (The STARD checklist for this diagnostic accuracy study is reported in the Additional file 2).

### Statistical analysis

Continuous normally distributed variables will be presented as means  ±  standard deviations (SD) and compared using the Student’s *t* test. Normality will be assessed using the Shapiro–Wilk test and visual inspection of quantile–quantile plots. Non-normally distributed data will be presented as medians, 1st and 3rd quartiles, and compared using the Wilcoxon rank-sum test. Categorical data will be compared between groups using the *χ*^2^ test, or Fisher’s exact test. Possible correlations between the patient outcomes and changes in LUS score will be examined using the Spearman's rank correlation coefficient. Receiver operating characteristic (ROC) curve analysis will be used to determine optimal cut-off values of LUS score for 30-day clinical deterioration (pulmonary, major cardiovascular events) and mortality. Youden’s index calculation will define the best cut-off value. Cox proportional-hazards models for mortality or clinical deterioration as endpoints allows for the calculation of hazard ratios (HR) of baseline LUS parameters. *P *values less than 0.05 will be considered to indicate statistical significance. Statistical analysis will be performed using the R environment (R Foundation for Statistical Computing, Vienna, Austria) and the appropriate packages.

### Power analysis

We estimate that 40% of patients have focal or diffuse B-lines with an altered LUS score—unexposed/exposed ratio: 1.5—and that 30% of patients have no B-lines, but could run into MACE with a relative risk of 1.5 [30, 31]. Considering that the rate of MACE in patients undergoing hip surgery is about 30%, we calculated a relative risk of 1.5. Therefore, we estimated that we would need a sample size of 877 patients in order to obtain 80% statistical power.

## Discussion

Patients with hip fracture encounter a significant risk of morbidity and mortality in the postoperative period [5, 30, 31]. The principal causes are cardiac-decompensated heart failure and myocardial infarction being the main diseases in this setting since the population is usually old (90% are aged  >  65 years) with the presence of underlying coronary artery disease [32, 33]. The number of patients undergoing this type of surgery is expected to increase over the coming years [31]. Thus, an extraordinary effort should be made to assess these patients in the preoperative setting. The ESA/ESC and ACC/ASA guidelines have attempted to provide the means to predict and quantify the risk of perioperative morbidity and mortality due to cardiac origin through a number of scoring systems [3, 4, 12]. However, the RCRI has shown only moderately accuracy in predicting the overall patient mortality risk as to the other scores [11]. That said, the evaluation of physical status with METS endorsed by ESA/ESC and ACC/ASA, is currently being assessed as part of a large observational study to ascertain its utility, both in self-reporting as well as METS testing [14]. The results of the “MET-repair” study are expected to be presented soon. The Canadian guidelines on perioperative evaluation have assigned a central role to BNP and Pro-BNP in their algorithm; however, while natriuretic peptides have been shown to have high negative predictive value, they do not have high positive predictive value [15]. Indeed, one study reported that clinicians perceive BNP to increase postoperative risk in only 66% of the patients [34].

More and more anesthesiologists are turning to LU [35]. Some studies reveal LU to have higher diagnostic accuracy than chest X-rays in the perioperative setting for the direct assessment of the most frequent lung diseases, such as pleural effusion, consolidation, and interstitial syndrome [20, 36]. Another recent study in vascular surgery patients (the LUPPO study) indicated that LU could help evaluate “indirect” cardiac status at the bedside [19]. This is because B-lines tightly correlate with interstitial lung syndrome of cardiac origin [37]. On the contrary, the absence of multiple B-lines excludes pulmonary edema with a high negative predictive value [24, 38]. Furthermore, an expert consensus document reporting a checklist for the quantification of pulmonary congestion by LU in heart failure reports that LU can provide useful information [24]. Considering the above, we designed this multicenter observational study to assess the utility of LU in the management of frail elderly patients undergoing hip fracture repair.

Our study protocol has some limitations: first of all, the different participating centers may have different levels of experience in LU use and may operate different ultrasound machines for LU evaluation; second, distinct perioperative clinical management in different centers could influence patient clinical course and the incidence of complications. However, if we demonstrate LU to have the ability to predict postoperative cardiac and pulmonary complications in hip fracture patients, our observational study will help pave the way to the generation of future hypotheses and the design of further randomized clinical trials directed at improving patient outcome. To say that we acknowledge as a study limitation that patients with known acute heart failure or MACE at “priori” were excluded to have a pure vision about LU prediction.

## Conclusions

The study is currently in the enrollment phase. Due to the COVID-19 outbreak, we experienced a reduction in enrollment rates in March, April and May 2020. However, the study is expected to reach the required sample size by the end of 2021.

## Supplementary Information

**Additional file 1.** Study Case Report Form.

**Additional file 2.** STARD checklist.

## Data Availability

Not applicable.

## Abbreviations

ASA-PS: American Society of Anesthesiologists Physical Status
ESC: European Society of Cardiology
ESA: European Society of Anesthesiology
RCRI: Revised Cardiac Risk Index
NSQIP-MICA: National Surgical Quality Improvement Program Myocardial Infarction and Cardiac arrest
ACC: American College of Cardiology
AHA: American Heart Association
METS: Metabolic equivalents
BNP: Brain natriuretic peptide
NT-pro-BNP: Pro-natriuretic peptide
NPV: Negative predictive value
ACP: American College of Physicians
COPD: Chronic obstructive pulmonary disease
CEUR-FVG: Ethic Committed of Friuli Venezia Giulia
MACE: Major advance cardiac events
ESICM: European Society of Intensive Care Medicine
GDPR: General data protection regulation
CRF: Case report form
PI: Principal investigator
STARD: Standards for the Reporting of Diagnostic Accuracy Studies
POCUS: Point-of-care ultrasound statement
SD: Standard deviation
ROC: Receiver operating characteristic
HR: Hazard ratios
