# Supplementary material for: Association between preoperative evaluation with lung ultrasound and outcome in frail elderly patients undergoing orthopedic surgery for hip fractures: study protocol for an Italian multicenter observational prospective study (LUSHIP)
Source: Ultrasound J. 2021 Jun 7;13:30. doi: 10.1186/s13089-021-00230-w (PMC8184059; doi:10.1186/s13089-021-00230-w)
Supplement: Supplementary file 2 — Additional file 2. STARD checklist. [file 13089_2021_230_MOESM2_ESM.pdf]

Prot.N. 0018656 / P / GEN/ ARCS
Data: 25/06/2019 14:16:47

R.A. FRIULI VENEZIA GIULIA

ARCS – AZIENDA REGIONALE DI COORDINAMENTO PER LA SALUTE

SEDE LEGALE: Udine - Via Pozzuolo 330
Centralino: +39 0432/1438010
Fax: +39 0432/1438011
C.F./P.IVA 02948180308
PEC:arcs@certsanita.fvg.it

COMITATO ETICO UNICO REGIONALE
sede operativa CENTRO di RIFERIMENTO ONCOLOGICO
Istituto di ricovero e cura a carattere scientifico
Via Franco Gallini, 2 – 33081 AVIANO (PN) tel. 0434 659282

Egregio dr. Vetrugno Luigi
Sperimentatore responsabile
Azienda Sanitaria Universitaria Integrata di Udine - SOC Clinica di Anestesia e Rianimazione

Gent.ma Sig.ra Elda Cameranesi
Direttore SOC Affari Generali ASUI di Udine

OGGETTO: parere CEUR (seduta dd.04/06/2019 - odg 5.18) SCIOGLIMENTO RISERVE

ID studio	2817
Codice interno al centro	NON APPLICABILE
Tipologia studio	Osservazionale senza farmaco e dispositivo
Tipo studio	No Profit
Codice studio	LUSHIP
Titolo studio	"Utilità dell'ecografia polmonare nella valutazione preoperatoria dei pazienti da sottoporre a chirurgia ortopedica d'urgenza per frattura di femore"
Fase	NA
EudraCT	NA
Promotore	Azienda Sanitaria Universitaria Integrata di Udine – ASUI UD
CRO	NA
Centro - UO	Azienda Sanitaria Universitaria Integrata di Udine - SOC Clinica di Anestesia e Rianimazione
Centro coordinatore	SOC Clinica di Anestesia e Rianimazione A.S.U.I. Udine
Sperimentatore	Vetrugno Luigi

In riferimento allo studio indicato ed alla precedente comunicazione Prot. N. 17261 del 12.06.2019 di trasmissione del parere "*sub-condicione*", **si ritiene che la documentazione sotto indicata trasmessa a mezzo e-mail in data 21.06.2019:**

- Foglio informativo, modulo di consenso e modulo di revoca al trattamento dei dati personali v. 4.0 del 20.06.2019
- Foglio informativo, modulo di consenso e modulo di revoca partecipazione allo studio v. 4.0 del 20.06.2019
- Lettera al MMG v. 4.0 del 20.06.2019
- Scheda dati anonimizzata

sia conforme a quanto richiesto. Si conferma che le modifiche richieste dal Comitato sono state recepite, vengono pertanto sciolte le riserve e si conferma in via definitiva il Parere favorevole già espresso nella seduta del 04.06.2019:

Parere CEUR-2019-Os-101

Si ricorda che questo Comitato Etico dovrà essere informato della data di inizio e di conclusione dello studio, dell'eventuale sua sospensione od interruzione, per qualsiasi causa.

La presente lettera non costituisce autorizzazione all'avvio dello Studio, presso la Struttura Operativa in indirizzo, e che detta autorizzazione sarà formalizzata con successivo decreto della Direzione Aziendale presso cui si svolge lo studio.

Nel trasmettere un tanto, si coglie l'occasione per porgere i migliori saluti.

il Presidente del Comitato Etico Unico Regionale
Dott. Paolo Rossi
Firmato digitalmente

****

Il Comitato Etico Unico Regionale, istituito ai sensi del Decreto 8 febbraio 2013 e Legge Regionale n. 33 del 29.12.2015, con DGR 22 gennaio 2016 n. 73, Decreto n. 414/SPS del 31.03.2016, e Decreto 694/SPS del 31.05.2016, opera in osservanza a quanto previsto dal DM 15 luglio 1997, dalla Circolare n. 15 del 15 ottobre 2000, dal D. Lgs n. 211 del 24 giugno 2003, e nel rispetto delle norme di Buona Pratica Clinica (GCP-ICP).

struttura competente: Comitato Etico Unico Regionale del Friuli Venezia Giulia, via Pozzuolo 330 – 33100 Udine
responsabile del procedimento: Alessandra Bearz, telefono 0432.1438091, mail comitato.etico@arcs.sanita.fvg.it
responsabile dell'istruttoria: Caterina Comand, telefono 0432.1438092, mail caterina.comand@arcs.sanita.fvg.it
referente della comunicazione: Daniela Gerometta, telefono 0432.1438093, mail daniela.gerometta@arcs.sanita.fvg.it
