# Supplementary material for: Association between preoperative evaluation with lung ultrasound and outcome in frail elderly patients undergoing orthopedic surgery for hip fractures: study protocol for an Italian multicenter observational prospective study (LUSHIP)
Source: Ultrasound J. 2021 Jun 7;13:30. doi: 10.1186/s13089-021-00230-w (PMC8184059; doi:10.1186/s13089-021-00230-w)
Supplement: Supplementary file 1 — Additional file 1. Study Case Report Form. [file 13089_2021_230_MOESM1_ESM.pdf]

Prot.N. 0018656/P/GEN/ARCS
Date: 25/06/2019

R.A. FRIULI VENEZIA GIULIA

ARCS – REGIONAL HEALTH COORDINATION COMPANY
REGISTERED OFFICE: Udine – Via Pozzuolo 330
Switchboard: +39 0432/1438010
Fax: +39 0432/1438011
C.F./P.IVA 02948180308 PEC: arcs@certsanita.fvg.it

**SINGLE REGIONAL ETHICS COMMITTEE**
operational headquarters ONCOLOGICAL REFERENCE CENTER
Scientific hospitalization and treatment institute
Via Franco Gallini, 2 – 33081 AVIANO (PN) tel. 0434 659282

Dear dr. Vetrugno Luigi
Responsible investigator
Integrated University Health Authority of Udine
– SOC Anesthesia and Intensive Care Clinic

Dear Ms Elda Cameranesi
Director of SOC General Affairs ASUI of Udine

**SUBJECT: CEUR opinion (meeting dd.04 / 06/2019 – odg 5.18) DISSOLUTION OF RESERVES**

| | |
|---|---|
| Study ID | 2817 |
| Internal code in the center | NOT APPLICABLE |
| Type of study | Observational without drug and device |
| study type | No profit |
| Study code | LUSHIP |
| Study title | "Usefulness of lung ultrasound in the preoperative evaluation of patients to undergo emergency orthopedic surgery for fracture of the femur" |
| Phase | NA |
| EudraCT | NA |
| Promoter | Integrated University Health Authority of Udine – ASUI UD |
| CRO | NA |
| Center – OU | Integrated University Health Authority of Udine – SOC Anesthesia and Intensive Care Clinic |
| Coordinating Center | SOC Clinic of Anesthesia and Resuscitation A.S.U.I. Udine |
| Experimenter | Vetrugno Luigi |

With reference to the study indicated and the previous communication Prot. N. 17261 of 12.06.2019 for the transmission of the "sub-conditional" opinion, **it is believed that the documentation indicated below sent by e-mail on 21.06.2019:**

- Information sheet, consent form and revocation form for the processing of personal data v. 4.0 dated 20.06.2019
- Information sheet, consent form and withdrawal form for participation in the study v. 4.0 dated 20.06.2019
- Letter to the GP v. 4.0 dated 20.06.2019
- Anonymized data sheet

complies with the requirements. It is confirmed that the changes requested by the Committee have been accepted, the reservations are therefore dissolved and the favorable opinion already expressed in the meeting of 04.06.2019 is definitively confirmed:

**CEUR-2019-Os-101 Opinion**

Please note that this Ethics Committee must be informed of the start and end date of the study, of any suspension or interruption, for any reason.

This letter does not constitute authorization to start the Study, at the Operational Structure at address, and that this authorization will be formalized with a subsequent decree of the Company Management where the study is carried out.

In transmitting a bit, we take the opportunity to extend our best regards.

the President of the Single Regional Ethics Committee Dr. Paolo Rossi

Digitally signed

The Single Regional Ethics Committee, established pursuant to the Decree of 8 February 2013 and Regional Law no. 33 of 29.12.2015, with DGR 22 January 2016 n. 73, Decree n. 414 / SPS of 03.31.2016, and Decree 694 / SPS of 05.31.2016, operates in compliance with the provisions of the Ministerial Decree of 15 July 1997, of Circular no. 15 of 15 October 2000, by Legislative Decree n. 211 of June 24, 2003, and in compliance with the rules of Good Clinical Practice (GCP-ICP).

competent structure: Friuli Venezia Giulia Single Regional Ethics Committee, via Pozzuolo 330 - 33100 Udine responsible for the procedure: Alessandra Bearz, telephone 0432.1438091, mail committee.etico@arcs.sanita.fvg.it responsible for the investigation: Caterina Comand, telephone 0432.1438092, mail caterina.comand@arcs.sanita.fvg.it contact person for communication: Daniela Gerometta, telephone 0432.1438093, mail daniela.gerometta@arcs.sanita.fvg.it
